# Highly Dynamic Changes in the Activity and Regulation of Macroautophagy in Hearts Subjected to Increased Proteotoxic Stress

**DOI:** 10.3389/fphys.2019.00758

**Published:** 2019-06-26

**Authors:** Bo Pan, Megan T. Lewno, Penglong Wu, Xuejun Wang

**Affiliations:** ^1^ Division of Basic Biomedical Sciences, Sanford School of Medicine of the University of South Dakota, Vermillion, SD, United States; ^2^ Department of Pathophysiology, College of Basic Medical Sciences, Guangzhou Medical University, Guangzhou, China

**Keywords:** macroautophagy, TFEB, mTOR, proteinopathy, proteotoxicity, mice

## Abstract

Macroautophagy (referred to as autophagy hereafter) plays an important role in the quality control of cellular proteins and organelles. Transcription Factor EB (TFEB) globally activates the expression of genes in the autophagic-lysosomal pathway (ALP) to replenish lysosomes and ALP machineries. We previously reported that myocardial TFEB signaling was impaired in advanced cardiac proteinopathy; however, myocardial ALP status and TFEB activity at earlier stages of cardiac proteinopathy remain uncharacterized. Here a stable line of CryAB^R120G^ transgenic (R120G) and non-transgenic (NTG) littermate mice with cardiomyocyte-restricted overexpression of CryAB^R120G^ were used at 1, 3, and 6 months of age. At 1 month when no cardiac phenotypes other than aberrant protein aggregation are discernible, R120G mice displayed a 5-fold increase in myocardial LC3-II flux. Interestingly, the LC3-II flux increase co-existed with increases in mTOR complex 1 (mTORC1) activities as well as cytoplasmic, but not nuclear, TFEB proteins. This increase in cytoplasmic TFEB proteins occurred without any discernible alteration in TFEB activity as reflected by unchanged mRNA levels of representative TFEB target genes (*Mcoln1, M6pr, Sqstm1, Vps18*, and *Uvrag*). At 3 months of age when hypertrophy and diastolic malfunction start to develop, the LC3-II flux remained significantly increased but to a lesser degree (2-fold) than at 1 month. The LC3-II flux increase was associated with decreased mTORC1 activities and with increased nuclear TFEB proteins and TFEB activities. At 6 months of age when congestive heart failure is apparent in R120G mice, both LC3-II flux and TFEB activities were severely suppressed, while mTORC1 activity increased. We conclude that changes in both autophagy and TFEB signaling are highly dynamic during the progression of cardiac proteinopathy. Increases in autophagy occur before increases in TFEB activities but both increase in the compensatory stage of cardiac proteinopathy. Once congestive heart failure develops, both autophagy and TFEB signaling become impaired. Our results suggest that TFEB signaling is regulated by both mTORC1-dependent and -independent mechanisms in hearts subjected to increased proteotoxic stress. For therapeutic exploration, it will be important to test the effect of TFEB stimulation at the early, intermediate, and late stages of cardiac proteinopathy.

## Introduction

Even during normal protein synthesis, protein misfolding is inevitable, which can be further intensified by genetic and environmental factors that either interfere with normal protein folding or render native proteins misfolded. The deployment of misfolded proteins can be catastrophic to the cell; to avoid this detrimental progression, the cell has developed multi-layered mechanisms to minimize the level and toxicity of misfolded proteins within the cell. As a whole, these mechanisms are known as protein quality control (PQC) (Wang and Robbins, 2006). With the help from chaperones, a misfolded protein may be unfolded and then refolded correctly, thereby repairing the protein in the process. However, if this repair process fails, the misfolded protein, now referred to as a terminally misfolded protein, has one of two possible, immediate fates. The first one is prompt degradation by the ubiquitin-proteasome system (UPS) or the lysosome (Wang et al., 2013), which is a better, cleaner option for the cell overall. The toxic identity from this misfolded protein is immediately and permanently removed and therefore will no longer pose any further danger to the cell. However, if the misfolded proteins have overwhelmed or escaped the surveillance of chaperones and the UPS, the second and less desirable fate is to undergo aberrant protein aggregation (i.e., the process of forming aberrant aggregates) within the cell. Smaller protein aggregates, formed throughout the cytoplasm, are transported *via* the microtubule system to the microtubule organization center (AKA the cell center). Here the aggregates coalesce into larger structures, known as aggresomes, at the para-nuclear location. Aggresome formation is likely aimed to reduce the toxicity of these smaller aggregates by covering or burying the reactive hydrophobic motif of these misfolded proteins as well as to promote the removal of these aggregates by the autophagic-lysosomal pathway (ALP). However, this second fate is less favorable to the cell, because the process of aberrant protein aggregation results in an accrual of soluble and insoluble protein aggregates that will continually pose a threat to the cell (Wang and Robbins, 2014). The aberrant protein aggregates, especially the soluble and highly active intermediate species (e.g., pre-amyloid oligomers) are significantly toxic to the cell, causing cell dysfunction and ultimately cell death as well. Moreover, aberrant protein aggregation has been well demonstrated to impair proteasome proteolytic function. Impairing proteasome proteolytic function exacerbates the accumulation of misfolded proteins and protein aggregation, forming a vicious cycle (Bence et al., 2001; Chen et al., 2005; Liu et al., 2006). Suppression of aberrant protein aggregation with molecular and pharmacological chaperones or molecular tweezers protects the proteasome and breaks this cycle (Chen et al., 2005; Liu et al., 2006; Xu et al., 2017). Similarly, proteasome enhancement, *via* either genetic or pharmacological means, has also been shown to break the vicious cycle and protect against cardiac proteotoxic stress *in vitro* and *in vivo* (Li et al., 2011a,b; Ranek et al., 2013; Zhang et al., 2019). Although emerging evidence suggests that the UPS may participate in the disaggregation and degradation of aberrant protein aggregates (Prabhudesai et al., 2012; Lump et al., 2015; Xu et al., 2017; Cliffe et al., 2019), it is generally believed that the ALP is the primary mechanism for removing protein aggregates in the cytoplasm (Wang and Robbins, 2014). Indeed, activation of the ALP with various means has been shown to protect against proteotoxic stress *in vitro* and *in vivo* (Pattison et al., 2011; Bhuiyan et al., 2013; Pan et al., 2017; Singh et al., 2017; Ma et al., 2019). Along with degrading misfolded proteins, the ALP is also responsible for degrading defective organelles (e.g., damaged mitochondria), thereby playing an important quality control role in a bulkier manner than the UPS in PQC.

In muscle, especially striated muscle, the pathophysiological significance of increased proteotoxic stress (IPTS) and aberrant protein aggregation is exemplified by a heterogeneous group of disorders now referred to as myofibrillar myopathies, to which desmin-related myopathy (DRM) belongs. This group of diseases is linked to mutations in a number of genes, such as desmin (*DES*), αB-crystallin (*CRYAB*), myotilin (*MYOT*), filamin C (*FLNC*), Bcl-2–associated athanogene 3 (*BAG3*) or Z-band alternatively spliced PDZ-motif protein (*ZASP*) (Goldfarb and Dalakas, 2009). Many of the earlier identified gene mutations (e.g., mutations in *DES* and *CRYAB*) are better studied experimentally, especially in cardiac muscle (McLendon and Robbins, 2011). For example, stable transgenic mouse lines with cardiomyocyte-restricted transgenic overexpression of a human DRM-linked Arg120Gly missense mutant CRYAB (CryAB^R120G^) recapitulate most aspects of human desmin-related cardiomyopathy (DRC) (Vicart et al., 1998; Wang et al., 2001b), which is the main cause of DRM-related death in humans. Inarguably, this DRC mouse model (referred to as R120G mice hereafter) has played a significant role in investigating cardiac PQC as well as in experimental exploration of the pathogenic mechanisms and therapeutic strategies for cardiac IPTS (Sandri and Robbins, 2014). The R120G mice are the subject of the present study for the reason stated above and elaborated below.

Experimental studies have unequivocally demonstrated that IPTS, caused by genetic mutations or commonly acquired cardiomyopathies, is both sufficient to cause HF and essential for HF genesis as well (Wang et al., 2001a,b, 2011; Sanbe et al., 2004, 2007, 2009; Rajasekaran et al., 2007; Tannous et al., 2008b; McLendon and Robbins, 2011; Li et al., 2011a,b, 2017; Bhuiyan et al., 2013; Rajagopalan et al., 2013; Willis and Patterson, 2013; Meijering et al., 2015; Pan et al., 2017; Wang and Cui, 2017). There is a preponderance of evidence that IPTS resulting from increased production of misfolded proteins, decreased removal of misfolded proteins or both of these stated scenarios occurs in a large subset of heart failure in humans (Weekes et al., 2003; Sanbe et al., 2004; Gianni et al., 2010; Predmore et al., 2010; Day et al., 2013; Subramanian et al., 2015; Ahmed et al., 2016; Troncone et al., 2016; Rainer et al., 2018; Zech et al., 2019). However, no current clinical heart failure therapies are intended to target IPTS yet. This is at least in part, because our understanding of how cardiomyocytes or the heart handles IPTS remains incomplete. To help fill this critical gap, we performed the present study for a comprehensive characterization of the dynamic changes in myocardial autophagic activity and in its regulatory pathways in the R120G mice during the entire span of disease progression.

Using R120G-based IPTS mouse models, several reports have shown that autophagic activation is an adaptive response in DRC and that enhancing autophagy actually protects against DRC progression (Tannous et al., 2008a; Zheng et al., 2011; Bhuiyan et al., 2013; Pan et al., 2017; Ma et al., 2019). These reports described cardiac autophagic activation and impairment in the early and the advanced stage of DRC, respectively; however, the studies characterizing autophagic activities in the early stage of DRC made this conclusion without performing rigorous autophagic flux assays (Tannous et al., 2008a; Zheng et al., 2011). These earlier studies were conducted and published before most researchers in the field came to realize that an autophagic flux assay is essential for precisely assessing autophagic activities in a complex system (Gottlieb et al., 2015). To specifically address this issue, the present study has employed a widely used autophagic flux assay when determining myocardial status of the ALP at various disease stages of the R120G mice.

Transcription factor EB (TFEB) is a well-established master regulator for the ALP (Sardiello et al., 2009; Settembre et al., 2011). As a basic helix-loop-helix leucine zipper transcription factor of the MiT family (Rehli et al., 1999; Kuiper et al., 2003; Martini-Stoica et al., 2016), TFEB activates the transcription of and enables the coordinated expression of a network of genes that are pivotal to autophagosome formation and lysosomal genesis by binding to a promoter element known as *c*oordinated *l*ysosomal *e*xpression *a*nd *r*egulation (CLEAR) motif (Sardiello et al., 2009). The network of genes harboring the CLEAR motif in their promoters (also known as the CLEAR network) consists of genes involved in processes such as autophagy and lysosomal biogenesis (Palmieri et al., 2011). The activation of TFEB has been shown to stimulate ALP function, which enhances the clearance of misfolded or aggregation-prone proteins both in cellular models of protein misfolding and mouse models of neurodegenerative disease (Dehay et al., 2010; Tsunemi et al., 2012; Decressac et al., 2013). Moreover, we previously reported that overexpression of TFEB is capable of increasing autophagic flux and protects against IPTS in cultured cardiomyocytes; myocardial TFEB activities were markedly suppressed, which was associated with increased activation of the atypical serine/threonine kinase mechanistic target of rapamycin complex 1 (mTORC1), in the R120G mice at the advanced DRC stage (Pan et al., 2017). Using a similar but slightly different R120G mouse model (Rajasekaran et al., 2007), a more recent study confirms that autophagic flux and TFEB signaling are suppressed in mouse hearts with advanced DRC and has further demonstrated that overexpression of TFEB *via* viral vector-mediated gene therapy protects the DRC disease progression in mice (Ma et al., 2019). Thus, it is very likely that impairment of cardiac TFEB transactivation, perhaps due to increased mTORC1 activation, contributes to the pathogenesis of advanced DRC. However, so far it has not been documented whether TFEB activity changes during the earlier stages of DRC and, if so, how those changes correlate to mTORC1 signaling. It is important to answer these questions since the answers may guide a further search for effective and precise strategies to treat DRC at different stages of the disease.

In the present study, we investigated the dynamic changes of myocardial autophagic flux and the associated alteration of mTOR signaling as well as TFEB expression and activity in a *bona fide* mouse model of cardiac IPTS induced by cardiomyocyte-specific overexpression of CryAB^R120G^. We found that changes in autophagic flux, mTORC1 signaling, and TFEB expression and activity are highly dynamic during the full time course of DRC progression. Increases in myocardial autophagic flux occur earlier than increases in TFEB activities, while both occur in the compensatory stage of cardiac proteinopathy; both autophagic flux and the TFEB activities become impaired in the congestive heart failure stage. Our results also suggest that TFEB activation in hearts with IPTS is regulated by both mTORC1-dependent and -independent mechanisms. For therapeutic exploration, it will be important and interesting to test the effect of TFEB stimulation at both early, intermediate, and late stages of cardiac proteinopathy.

## Materials and Methods

### Animals

This study was carried out in accordance with the recommendations of the Guide for the Care and Use of Laboratory Animals (US Department of Health, Education, and Welfare, Department of Health and Human Services, NIH Publication 85-23). The protocol for the care and use of the animals in this study was approved by the University of South Dakota Institutional Animal Care and Use Committee. The creation and baseline characterization of the inbred FVB/N mice with transgenic overexpression of CryAB^R120G^ driven by the murine Myh6-promoter was previously described (Wang et al., 2001b). Mixed sex R120G and non-transgenic (NTG) littermate mice at 1, 3, and 6 months of age were used.

### Reagents

Triton X-100 (#T8532), ethylenediaminetetraacetic acid (EDTA: #E9884), phenylmethanesulfonyl fluoride (PMSF: P7626), bafilomycin A1 (BFA: #19-148), and fluoroshield with DAPI (#F6057) were purchased from Sigma-Aldrich (St. Louis, MO). TRIzol (#TR 118) was purchased from Molecular Research Center, Inc. (Cincinnati, OH). Tissue-Tek O.C.T (#4585) was purchased from Fisher Healthcare (Houston, TX). DreamTaq Green DNA Polymerase (#EP0712) was purchased from ThemoFisher Scientific (Waltham, MA).

### Total Protein Extraction and Western Blot Analysis

Proteins were extracted from ventricular myocardium using a buffer (pH 6.8) containing 1.0 M Tris-HCl, 10% SDS, 10% glycerol, and a complete protease inhibitor cocktail. A cocktail of protease inhibitors (#T-2496, AG. Scientific, San Diego, CA) was added to the extraction buffer to inhibit protein degradation. Protein concentration was determined using bicinchoninic acid reagents (#23225, ThemoFisher Scientific, Waltham, MA). Equal amounts of proteins were fractionated *via* 8–14% SDS-PAGE. The separated proteins were transferred onto PVDF membranes, which were then sequentially subjected to blocking, incubating with primary antibodies against the proteins of interest, washing with TBS-T to remove unbound primary antibodies, incubating with horseradish peroxidase (HRP) conjugated goat anti-mouse IgG (#115-035-003) or anti-rabbit IgG (#111-035-003) secondary antibodies (Jackson ImmunoResearch Laboratories, West Grove, PA), and washing to remove unbound antibodies. The secondary antibodies bound to the PVDF membrane were then detected using enhanced chemiluminescence reagents, which was then digitally imaged and analyzed using the ChemiDoc™ MP imaging system and associated software (Bio-Rad, Hercules, CA) as we previously reported (Pan et al., 2017). The following primary antibodies were used: Anti-GAPDH (#G8795, Sigma-Aldrich; 1:1,000), LC3B (#2775, Cell Signaling; 1:1,000), Anti-Alpha B Crystallin (#ab13496, Abcam; 1:1,000), mTOR (#2983, Cell Signaling; 1:1,000), Phospho-mTOR (Ser2481) (#2974, Cell Signaling; 1:1,000), p70 S6 kinase (#2708, Cell Signaling; 1:1,000), Phospho-p70 S6 kinase (Thr389) (#9234, Cell Signaling; 1:1,000), 4E-BP1 (#9644, Cell Signaling; 1:1,000), Phospho-4E-BP1 (Thr37/46) (#2855, Cell Signaling; 1:1,000), and TFEB (#A303-673A, Bethyl Laboratories, Inc.; 1:1,000). For a loading control, stain-free total protein imaging technology was used as previously described (Zhang et al., 2019).

### Cytoplasmic and Nuclear Fractionation of Ventricular Myocardium

Cytoplasmic and nuclear proteins were extracted using the Nuclear Extraction Kit (#P504, 101Bio, Mountain View, CA) according to the manual provided by the manufacturer. In brief, tissue (30 mg) was weighed, washed with PBS, and centrifuged for 1 min at 3,000 rpm; the supernatant was discarded. The cytoplasmic extraction buffer was then added to the tissue. The tissue was homogenized using a micro-grinder and then incubated on ice for 5 min, during which the homogenates were vortexed vigorously for 15 s every minute and then centrifuged at 4°C for 5 min at 12,000 rpm. The resultant supernatant was the cytoplasmic protein extract, which was then transferred to a pre-chilled 1.5 ml tube. The pellet was then washed 2 times with PBS before adding appropriate amounts of nuclear extraction buffer. This was then incubated on ice and vortexed vigorously for 15 s every minute for 4 min. The nuclear extract mixture was then transferred to a pre-chilled filter cartridge with a collection tube and centrifuged at 12,000 rpm for 30 s at 4°C. The filtrate was collected as the nuclear extract. Both the cytoplasmic and the nuclear extracts were stored at −80°C until use.

### Myocardial LC3-II Flux Assay

For the assessment of autophagic flux in the heart, mice were subjected to an intraperitoneal injection with bafilomycin A1 (BFA, 3 μmol/kg body weight), and hearts were collected 1 h later. LC3-II protein levels were determined using western blot analysis. The LC3-II flux presented here refers to the net amount of LC3-II accumulated by the BFA-mediated lysosomal inhibition. Mathematically, it is calculated by subtracting the mean value of the LC3-II levels (GAPDH normalized) of a BFA-treated sample with the mean value of the LC3-II levels (GAPDH normalized) of the DMSO-treated samples from the same group (Wu et al., 2017).

### RNA Isolation, Complementary DNA Synthesis, and Quantitative PCR

Total RNA was isolated as previously described (Zhang et al., 2016). cDNA synthesis was performed using a kit (#4374966, ThemoFisher Scientific, Waltham, MA) by following manufacturer’s instructions. The total volume for PCR was 50 μl, comprised of 40 μl water, 1 μl of each primer (10 μM), 5 μl 10× buffer mix with Mgcl_2_, 1 μl Taq DNA polymerase, and 1 μl cDNA. The PCR amplification was as follows: denaturation at 95°C for 5 min, 24–28 PCR cycles of 95°C for 20 s, 55–62°C for 20 s, 72°C for 30 s, followed by 1 cycle at 72°C for 10 min. GAPDH was used as a housekeeping gene and was used to normalize the PCR product levels of other genes, so that expression levels among different groups could be analyzed. Sequences of the specific primers that were designed by VectorNTI Advance 10 software (Pan et al., 2014) were previously reported (Pan et al., 2017).

### Statistical Analysis

All continuous variables are presented as a mean ± SEM unless otherwise indicated. Differences between two groups were evaluated for statistical significance using a two-tailed unpaired *t*-test. When the differences among three or more groups were evaluated, one-way ANOVA was used; when appropriate, two-way ANOVA followed by the Tukey test for pairwise comparisons was performed. *p* < 0.05 is considered statistically significant.

## Results

### Dynamic Changes of Myocardial Autophagic Flux in Mice With CryAB^R120G^-Based IPTS

**Figure 1:**
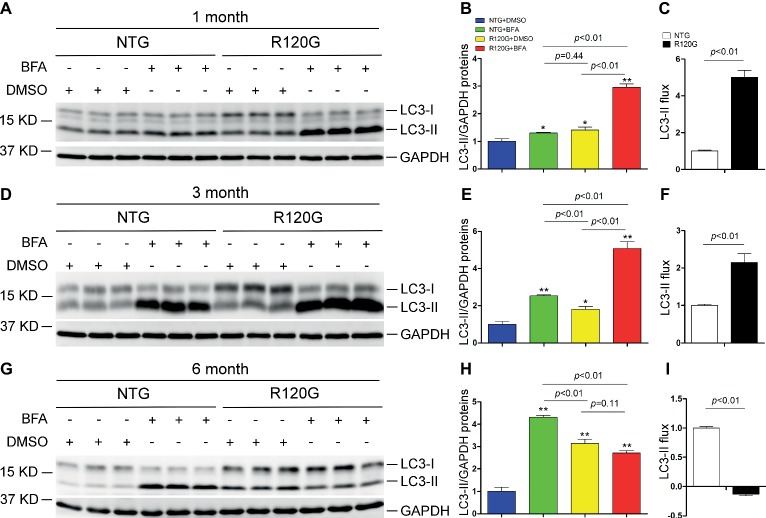
Change in myocardial LC3-II flux in CryAB^R120G^ transgenic mice at 1, 3 and 6 months of age. CryAB^R120G^ transgenic (R120G) and non-transgenic (NTG) littermate mice at 1, 3 or 6 months of age were treated with bafilomycin A1 (BFA, 3 μmol/kg) or DMSO (vehicle control), and their ventricular myocardial samples were collected for extraction of total proteins. The extracted proteins were used for western blot analyses for LC3 and GAPDH, a loading control. Shown are the representative images **(A,D,G)**, a summary of LC3-II densitometry data **(B,E,H)**, and the LC3-II flux **(C,F,I)** derived from the data presented in **(B), (E)** and **(H)**, respectively. ^*^p < 0.05, ^**^p < 0.01 vs. NTG + DMSO; *n* = 3 mice per group; two-way **(B,E,H)** or one-way **(C,F,I)** ANOVA followed by Tukey test for pairwise comparison.

Prior studies have consistently shown decreases in myocardial autophagic flux in mice with advanced DRC induced by cardiomyocyte-restricted transgenic overexpression of CryAB^R120G^ (Pan et al., 2017; Ma et al., 2019), a human disease-linked misfolded protein (Vicart et al., 1998); however, to date the status of autophagy in the heart in the early and intermediate stages of cardiac proteinopathy have not been rigorously examined. The disease progression of the R120G mice displays well-documented distinct stages. At 1 month of age (1 m), other than aberrant protein aggregation, these mice do not have any discernible cardiac morphometric and functional abnormalities; however, at 3 months of age (3 m), these mice develop cardiac hypertrophy and diastolic malfunction or heart failure with preserved ejection fraction (HFpEF) (Zhang et al., 2019); by 6 months of age (6 m), dilated cardiomyopathy and heart failure with reduced ejection fraction (HFrEF) become evident in these mice and result in their premature death between 6 and 7 months of age (Wang et al., 2001b). Hence, we determined the time course of autophagic flux changes at these three distinct stages using the *in vivo* LC3-II flux assay (Figure 1). Since LC3-II stays in the membrane of autophagosomes throughout their entire lifespan, changes in LC3-II protein levels are widely used as an indicator of changes in the abundance of autophagosomes in cells or tissues (Wang and Cui, 2017). For the LC3-II flux assay, ventricular myocardial samples were collected 1 h after the injection of bafilomycin A1 (BFA, a specific inhibitor of vacuolar-type proton ATPase) to inhibit lysosomal degradation of autophagosomes. Western blot analyses revealed that myocardial LC3-II protein levels in the vehicle control (DMSO) CryAB^R120G^ mice were discernibly higher than that of the vehicle control sex-matched NTG littermates at all three time points (i.e., 1, 3, and 6 m); and the differential between the R120G and NTG groups rises progressively from 1 to 6 m (Figure 1). More importantly, lysosomal inhibition with BFA led to a significantly greater LC3-II accumulation in the R120G mice than in NTG littermates at both 1 and 3 m, indicative of a significant increase in myocardial LC3-II flux in the R120G mice at these earlier time points (Figures 1A–F); however, at 6 m when cardiac proteinopathy is advanced in these mice, the BFA treatment no longer increased myocardial LC3-II in the R120G mice as it did with the NTG littermates (Figures 1G–I). This collection of data reveals for the first time that the alterations of myocardial autophagic flux in mice with cardiac proteinopathy are highly dynamic; LC3-II flux is significantly increased at the earlier stages of the disease, but it becomes severely impaired as the disease progresses to the more advanced stage. The significant increase of the basal LC3-II levels and the drastically decreased LC3-II flux in the R120G mice at 6 m indicate a severe impairment in autophagosome removal by lysosomes.

Notably, myocardial LC3-II flux in the NTG control mice showed a progression increase from 1 to 6 m (Figure 1), suggesting that cardiac autophagic activities undergo a steady upregulation as the animal grows up during the postnatal development.

### Changes in Myocardial mTOR and TFEB Signaling in the R120G Mice at 1 m

Both mTORC1 and TFEB play a major role in the regulation of ALP activities in the cell, but their activation has opposing effects on ALP activities. TFEB appears to be the master transcription factor for replenishing ALP machinery, essential to sustaining ALP activities; however, mTORC1 generally suppresses autophagy *via* various mechanisms, including phosphorylating TFEB at multiple residues to sequester TFEB in the cytoplasm, thereby preventing TFEB from entering the nucleus (Wang and Cui, 2017). To explore the molecular mechanisms governing the dynamic changes of autophagy in the proteinopathic R120G mouse hearts, we examined the activation status of both mTORC1 and TFEB signaling at the three representative time points.

**Figure 2:**
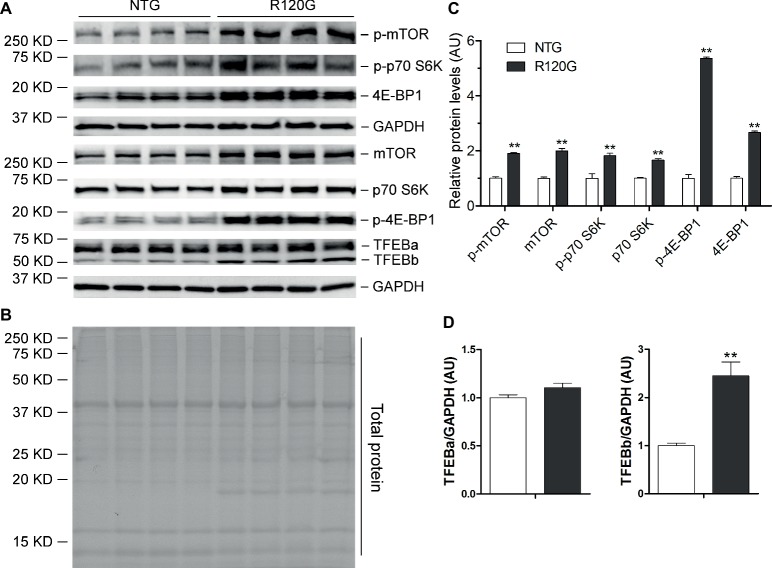
Western blot analyses for the indicated proteins of the mTORC1 and TFEB signaling pathways in the ventricular myocardium from the R120G and NTG littermate mice at 1 month of age. Shown are representative images of western blot for total mTOR (mTOR) and Ser2481-phosphorylated mTOR (p-mTOR), total p70 S6 kinase (p70 S6K) and Thr389-phosphorylated p70 S6K (p-p70 S6K), total 4E-BP1 and Thr37/46-phosphorylated 4E-BP1 (p-4E-BP1), TFEB as well as GAPDH (a loading control) **(A)**, a representative stain-free total protein image on a PVDF membrane that was used for immunoblotting **(B)**, and the pooled densitometry data **(C,D)**. ^**^p < 0.01 vs. NTG; *n* = 4 mice per group; two-tailed unpaired *t*-test.

**Figure 3:**
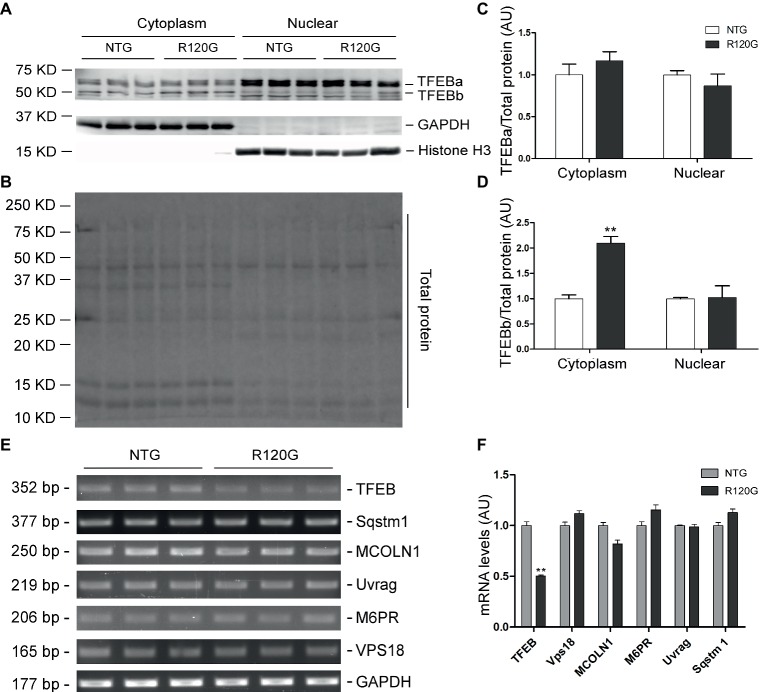
Western blot analyses for subcellular distribution of myocardial TFEB proteins and RT-PCR analyses of representative target genes of TFEB in the R120G and NTG littermate mice at 1 month of age. **(A–D)** Cytoplasmic and nuclear protein extracts were subjected to western blot analyses for the indicated proteins. GAPDH and Histone H3 were probed as a cytoplasmic and nuclear loading control, respectively. Shown are the representative images **(A)**, a representative stain-free total protein image of a PVDF membrane used for protein normalization **(B)**, pooled densitometry data of subcellular distribution of TFEB proteins **(C,D). (E)** and **(F)**, representative PCR images of mRNA levels of TFEB and the indicated representative target genes of TFEB **(E)** and the pooled densitometry data **(F)**. ^**^p < 0.01 vs. NTG; *n* = 3 mice per group; two-tailed unpaired *t*-test.

Despite any evidence of cardiac hypertrophy in the R120G mice at 1 m (Wang et al., 2001b), myocardial phosphorylated forms of mTOR and of known mTORC1 targets, such as p70 S6 kinase (p70 S6K) and 4E-BP1, as well as their total protein levels were markedly increased, indicative of increased mTORC1 activity, as compared with their NTG littermates at 1 m (Figures 2A–C). As we reported previously (Pan et al., 2017), two main forms of TFEB proteins (TFEBa and TFEBb) were detectable in mouse ventricles (Figure 2A). Compared with NTG mice, a significant increase of TFEBb were detected in the R120G mice at 1 m, while no discernible changes occurred in TFEBa (Figure 2D). Western blot analyses of subcellular fractions showed that the increased TFEBb resided primarily in the cytoplasm. The nuclear fraction of TFEB was comparable between the R120G and NTG mice at this time point (Figures 3A–D). This comparability suggests that the nuclear translocation for activation of TFEB is not altered by TG expression of CryAB^R120G^ at this early time point. This is further supported by the data that shows myocardial mRNA levels of representative TFEB target genes (*Mcoln1, M6pr, Sqstm1, Vps18*, and *Uvrag*) were also comparable between R120G and NTG mice (Figures 3E,F). These findings strongly indicate that TFEB activity remains unchanged in the R120G hearts at 1 m, which contradicts both the increased mTORC1 activity and increased autophagic flux. mTORC1 activity is expected to inhibit TFEB activity, while increased autophagic flux is often associated with TFEB activation.

### Changes in Myocardial mTOR and TFEB Signaling in the R120G Mice at 3 m

**Figure 4:**
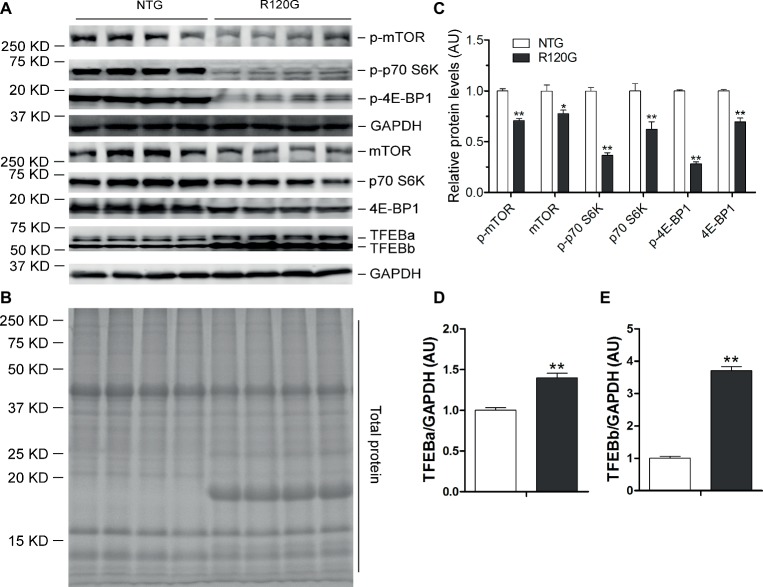
Western blot analyses for the indicated proteins of the mTORC1 and TFEB signaling pathways in the ventricular myocardium from the R120G and NTG littermate mice at 3 months of age. Shown are representative images of western blot for total mTOR (mTOR) and Ser2481-phosphorylated mTOR (p-mTOR), total p70 S6 kinase (p70 S6K) and Thr389-phosphorylated p70 S6K (p-p70 S6K), total 4E-BP1 and Thr37/46-phosphorylated 4E-BP1 (p-4E-BP1), TFEB as well as GAPDH (a loading control) **(A)**, a representative stain-free total protein image on a PVDF membrane that was used for immunoblotting **(B)**, and the pooled densitometry data **(C–E)**. ^*^p < 0.05, ^**^p < 0.01 vs. NTG; *n* = 4 mice per group; two-tailed unpaired *t*-test.

**Figure 5:**
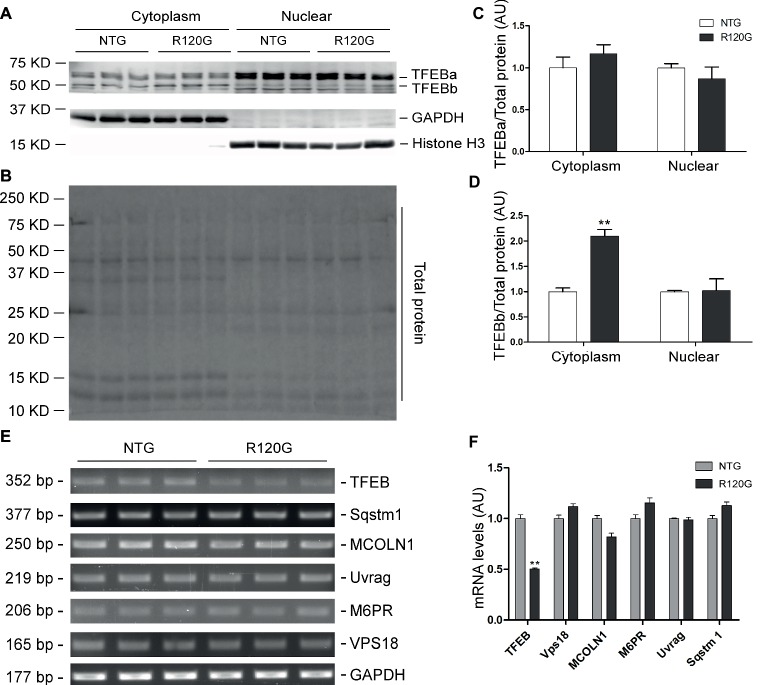
Western blot analyses for subcellular distribution of myocardial TFEB proteins and RT-PCR analyses of representative target genes of TFEB in the R120G and NTG littermate mice at 3 months of age. **(A–D)** Cytoplasmic and nuclear protein extracts were subjected to western blot analyses for the indicated proteins. GAPDH and Histone H3 were probed as a cytoplasmic and nuclear loading control, respectively. Shown are the representative images **(A)**, a representative stain-free total protein image of a PVDF membrane used for protein normalization **(B)**, pooled densitometry data of subcellular distribution of TFEB proteins **(C,D). (E)** and **(F)**, representative PCR images of mRNA levels of TFEB and the indicated representative target genes of TFEB **(E)** and the pooled densitometry data **(F)**. ^**^p < 0.01 vs. NTG; *n* = 3 mice per group; two-tailed unpaired *t*-test.

In stark contrast to 1 m, myocardial mTORC1 activity was markedly suppressed in the R120G mice at 3 m as evidenced by significant, across-the-board decreases in both the total and the phosphorylated forms of mTOR, p70 S6K, and 4E-BP1 compared with their NTG littermates (Figures 4A–C). At this time point, myocardial protein levels of both TFEBa and TFEBb were significantly higher in the R120G mice than in NTG mice (Figures 4A,D). Western blot analyses further showed that both TFEBa and TFEBb were markedly increased in the nuclear fraction of the R120G hearts. In the cytoplasmic fraction of the R120G hearts, TFEBa is decreased, while TFEBb is increased compared with their NTG littermates (Figures 5A–D), indicating that nuclear translocation of TFEBs is remarkably increased in the proteinopathic mouse hearts at this time point. Consistently, the myocardial steady state mRNA levels of the examined TFEB target genes, including TFEB itself, were all significantly increased in the R120G mice compared with their NTG littermates (Figures 5E,F), demonstrating that TFEB transactivation activities are upregulated at this stage. This is consistent with the decreased activity of mTORC1 and increased nuclear translocation of TFEB proteins in the proteinopathic hearts. Very likely, the upregulated TFEB activation contributes to sustaining the increased autophagic flux from 1 to 3 m in the proteinopathic mouse hearts.

### Changes in Myocardial mTOR and TFEB Signaling in the R120G Mice at 6 m

**Figure 6:**
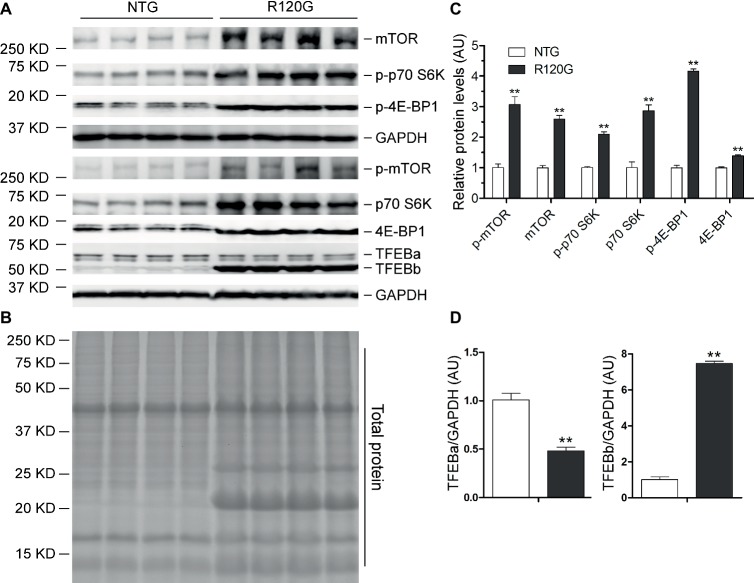
Western blot analyses for the indicated proteins of the mTORC1 and TFEB signaling pathways in the ventricular myocardium from the R120G and NTG littermate mice at 6 months of age. Shown are representative images of western blot for total mTOR (mTOR) and Ser2481-phosphorylated mTOR (p-mTOR), total p70 S6 kinase (p70 S6K) and Thr389-phosphorylated p70 S6K (p-p70 S6K), total 4E-BP1 and Thr37/46-phosphorylated 4E-BP1 (p-4E-BP1), TFEB as well as GAPDH (a loading control) **(A)**, a representative stain-free total protein image on a PVDF membrane that was used for immunoblotting **(B)**, and the pooled densitometry data **(C,D)**. ^**^p < 0.01 vs. NTG; *n* = 4 mice per group; two-tailed unpaired *t*-test.

**Figure 7:**
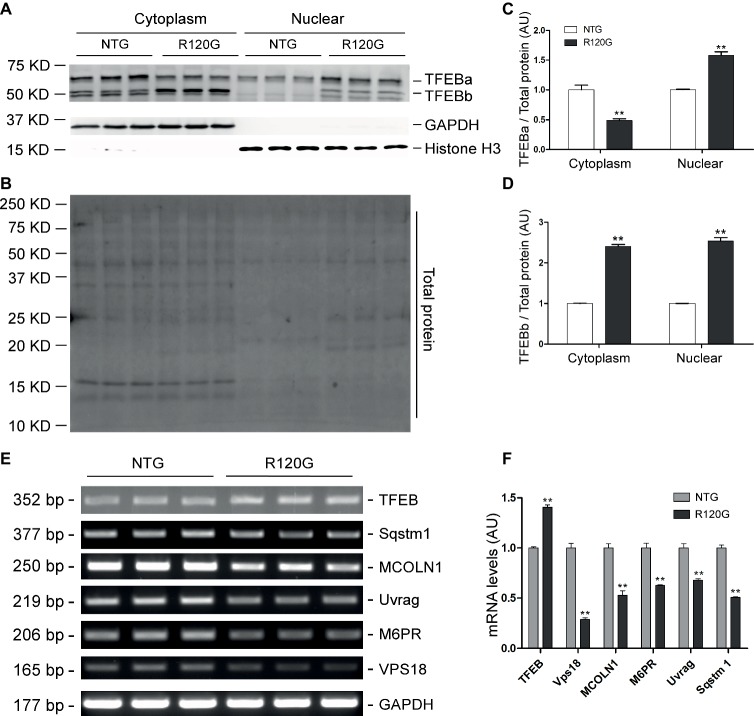
Western blot analyses for subcellular distribution of myocardial TFEB proteins and RT-PCR analyses of representative target genes of TFEB in the R120G and NTG littermate mice at 6 months of age. **(A–D)** Cytoplasmic and nuclear protein extracts were subjected to western blot analyses for the indicated proteins. GAPDH and Histone H3 were probed as a cytoplasmic and nuclear loading control, respectively. Shown are the representative images **(A)**, a representative stain-free total protein image of a PVDF membrane used for protein normalization **(B)**, pooled densitometry data of subcellular distribution of TFEB proteins **(C,D). (E)** and **(F)**, representative PCR images of mRNA levels of TFEB and the indicated representative target genes of TFEB **(E)** and the pooled densitometry data **(F)**. ^**^p < 0.01 vs. NTG; *n* = 3 mice per group; two-tailed unpaired *t*-test.

In agreement with our prior report (Pan et al., 2017), myocardial mTORC1 signaling was remarkably upregulated in the R120G mice compared with their NTG littermates at 6 m as evidenced by increased levels of both the total and the phosphorylated forms of mTOR, p70 S6K, and 4E-BP1 (Figures 6A–C). Interestingly, differential changes between TFEBa and TFEBb protein levels were observed in the R120G mice at 6 m; TFEBa was lower, while TFEBb was higher in the R120G than the NTG littermates at 6 m (Figures 6A,D). Western blot analyses of TFEB in the cytoplasmic and nuclear fractions revealed that both TFEBa and TFEBb were significantly increased in the nuclear fraction and, in the cytoplasmic fraction, TFEBa was decreased, while TFEBb was increased in the R120G hearts compared with their NTG littermates (Figures 7A–D). RT-PCR analyses showed that the myocardial steady state mRNA levels of all representative TFEB target genes (*Mcoln1, M6pr, Sqstm1, Vps18*, and *Uvrag*) were significantly lower, besides TFEB in itself, in the R120G mice than in NTG littermates (Figures 7E,F), indicating that TFEB activity is suppressed in the R120G hearts at this advanced stage of the disease.

## Discussion

**Figure 8:**
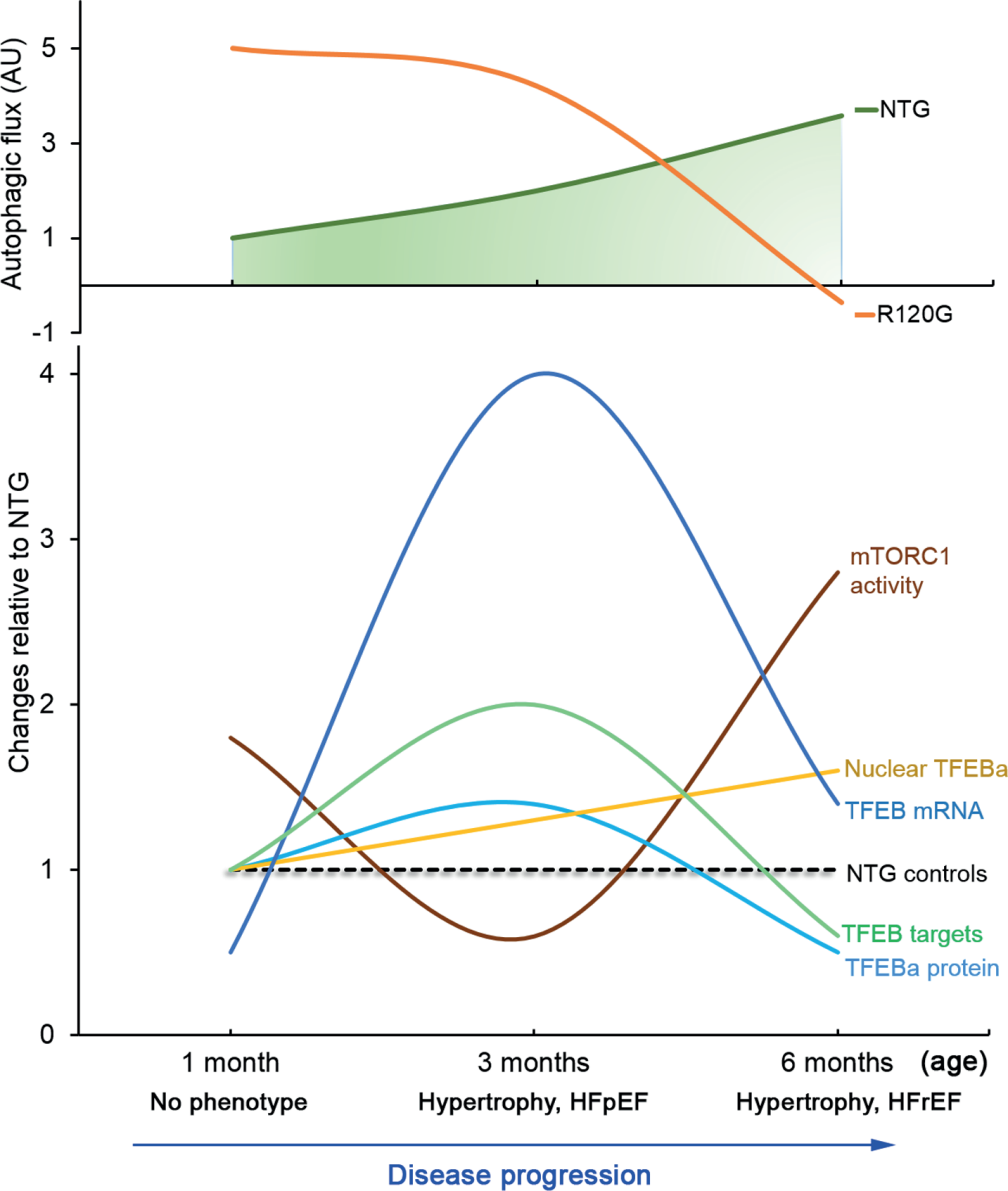
A schematic summery of the dynamic changes in myocardial autophagic flux as well as in mTORC1 and TFEB signaling during DRC progression in the CryAB^R120G^ transgenic mice (R120G) compared with the NTG littermates. The numerical value of mTORC1 activity is the average fold changes of p-mTOR, p-p70S16K, and p-4E-BP1. The numerical value of TFEB targets is the average fold changes of all representative target genes examined. HFpEF, heart failure with preserved ejection fraction; HFrEF, heart failure with reduced ejection fraction.

Increased proteotoxic stress has been observed in a majority of animal models for both common and rare forms of heart disease. IPTS has been shown to actually contribute to the progression of these diseases to heart failure; it is also implicated in the genesis of a large subset of heart failures in humans as well (Sandri and Robbins, 2014). By globally regulating lysosomal genesis and the ALP, TFEB is pivotal for the cell to manage IPTS (Wang and Cui, 2017); hence, a better understanding of the full time course of myocardial TFEB expression and activity changes in a *bona fide* animal model of cardiac IPTS should guide the effort for the development of more precise therapeutic strategies for heart disease with IPTS, to which a large subset of heart failure belongs (Sandri and Robbins, 2014; Zech et al., 2019). Prior studies on the changes in TFEB and autophagic activities induced by cardiac proteinopathy were focused only on the advanced stage of the disease (Pan et al., 2017; Ma et al., 2019). This leaves the question of how TFEB expression and activation responds to cardiac IPTS only partially addressed. The present study has comprehensively determined the full time course of the dynamic changes in myocardial autophagic activities, mTOR signaling, and TFEB expression and transactivation activity in the R120G mice (Figure 8), a well-characterized and widely used mouse model of cardiac IPTS. Here we have confirmed the previously reported increase in autophagic activity at the early and intermediate stages (1 and 3 m) and the mTORC1 activation associated TFEB and ALP suppression at the advanced stage (6 m) of the disease. Moreover, we have discovered that not only does the degree of autophagic flux increase in the R120G mice differ between the early (1 m) and intermediate (3 m) stages but also the increases in myocardial autophagic activity at the two time points are associated with differential changes in mTORC1 and TFEB activities. This indicates that increased autophagy at the compensatory stages (1 and 3 m) of the disease is underlined by both TFEB and mTORC1-dependent and -independent mechanisms.

The significance of the present study is also underscored by that the R120G mice employed here not only are a widely used animal model of human DRC that recapitulates most cardiac manifestations of human DRM but also represent valuable model for *in vivo* studies on how the heart responds to IPTS, an underappreciated cardiac pathogenic factor that has been implicated in a large subset of human heart failure (Sanbe et al., 2004; Gianni et al., 2010). For example, pre-amyloid oligomers, a prominent indicator of aberrant protein aggregation and impaired PQC, were detected in great abundance in both the R120G mouse hearts and a large subset of failing human hearts (Sanbe et al., 2004); in both cases, alterations in desmin proteins are associated with the increased pre-amyloid oligomers and cardiac malfunction (Rainer et al., 2018).

### Myocardial Autophagy Is Increased at the Compensatory Stage of Cardiac Proteinopathy and This Increase Is Mediated by TFEB and mTORC1 Independent and Dependent Mechanisms

mTORC1 integrates many extracellular and intracellular cues, including growth factors, inflammatory cytokines, energy status, nutrient conditions, amino acids, redox states, and lysosomal stress and, by virtue of altering its kinase activities, regulates various major cellular processes including protein synthesis (e.g., *via* phosphorylating p70-S6 kinase and 4E-BP1), metabolism, inflammation, and the ALP (Kim and Guan, 2015). mTORC1 negatively regulates ALP activities in multiple ways, including phosphorylation and thereby inhibition of TFEB (Vega-Rubin-de-Celis et al., 2017). At least a portion of TFEB and mTORC1 are localized on the membrane of lysosomes (Vega-Rubin-de-Celis et al., 2017). Under normal nutrient-rich conditions, mTORC1 phosphorylates TFEB at Ser211 (Martina et al., 2012; Roczniak-Ferguson et al., 2012), Ser142 (Settembre et al., 2012), and Ser122 (Vega-Rubin-de-Celis et al., 2017), which triggers the 14-3-3 binding of TFEB and the retaining of TFEB in the cytoplasm, thereby suppressing its nuclear translocation (Bajaj et al., 2019). Conversely, mTORC1 inhibition by starvation or lysosomal stress terminates mTORC1-mediated phosphorylation and suppression of TFEB, allowing the translocation of TFEB into the nucleus to activate the expression of the CLEAR network genes to sustain increased ALP activities (Wang and Cui, 2017).

Here, we found that increased myocardial autophagic flux (Figures 1D–F) in the R120G mice at 3 m was associated with inactivation of mTORC1 signaling as reflected by marked decreases in p-mTOR, mTOR, p-70S6K, and p-4E-BP1 (Figure 4) and with increased TFEB transactivation activities as evidenced by increased nuclear translocation of TFEB (Figures 5A–D) and increased mRNA levels of representative TFEB target genes (Figures 5E,F). These changes perfectly fit the model at this stage. Cardiomyocytes have sensed the extraordinarily high lysosomal stress caused by accumulation of aberrant CryAB aggregates, thus mTORC1 is shut down, which in turn relieves mTORC1’s inhibition on TFEB. This then allows TFEB to translocate into the nucleus where it activates the transcription of genes from the CLEAR network, increasing autophagic flux. Hence, increased ALP activity at 3 m is likely attributable to mTORC1 inactivation and TFEB activation.

Surprisingly, changes of myocardial autophagic flux, TFEB activity, and mTORC1 signaling in the R120G mice at 1 m did not seem to follow the known functional relationship among the three. At this very early stage of the disease and also the young age of the animals, intracellular CryAB aberrant aggregates are clearly detectable but cardiac hypertrophy and malfunction are not apparent yet in the R120G mice (Wang et al., 2001b). Myocardial autophagic flux in the R120G mice was as high as ~5 times that of their NTG littermates (Figures 1A–C), a much greater increase than the increase observed at 3 m (~2 times); however, this remarkable increase was associated with increased mTORC1 activation (Figure 2) and virtually unchanged TFEB activities (Figure 3). How to explain this phenomenon? We pose that at 1 m, dictated by normal cardiac growth, mTORC1 is highly activated which would inhibit TFEB activity. This could be countered by stimulated TFEB activation by the increased demand on ALP machinery from increased autophagic activity, rendering the TFEB activity in the R120G mice comparable to NTG mice at 1 m when the baseline autophagic flux is rather low (Figure 1A). Hence, the baseline TFEB activity is sufficient to sustain the increased autophagy at this point. Since mTORC1 activity is not decreased in the R120G hearts at 1 m, the autophagic activation is apparently mTORC1-independent. It will be interesting and important to identify the pathway linking cardiac IPTS to autophagic activation at this early stage.

### Myocardial Autophagy and TFEB Signaling Are Impaired at the Decompensated Stage of Cardiac Proteinopathy but mTORC1 Activation Might Not Be the Cause

Dysregulation of the ALP has been observed in a variety of cardiomyopathies (Zech et al., 2019). ALP activation by genetic means (e.g., overexpression of ATG7 or TFEB) (Bhuiyan et al., 2013; Ma et al., 2019), calorie restriction (Singh et al., 2017; Ma et al., 2019), or pharmacological agents (Eisenberg et al., 2016; Singh et al., 2017; Sciarretta et al., 2018; Carmona-Gutierrez et al., 2019) has mostly been shown beneficial to the heart although there is some evidence that excessive activation of autophagy could also be detrimental to the heart in conditions such as reperfusion injury (Sciarretta et al., 2018). We confirmed an increase in mTORC1 activity (Figure 6) and a decrease in myocardial TFEB transactivation activity in the R120G mice at 6 m (Figure 7) as we previously reported (Pan et al., 2017). Consistent with these changes, a marked decrease in myocardial autophagic flux is unveiled by a LC3-II flux assay for the first time in the R120G mice at 6 m (Figures 1G–I). Apparently, this decreased TFEB activity and autophagic impairment play an important pathogenic role in the advanced stage of cardiac proteinopathy because rescue effects from either viral delivery of TFEB to the heart at the advanced disease stage or germ-line transgenic enhancement of cardiac autophagy on mouse models of CryAB^R120G^-based proteinopathy had been reported by others (Bhuiyan et al., 2013; Ma et al., 2019). Therefore, it is important to further delineate the cause underlying the suppressed TFEB transactivation activity in the R120G hearts. The co-existed activation of the mTORC1 signaling (Figure 6) certainly is the primary suspect, because mTORC1 is known to negatively regulate TFEB signaling. However, two other lines of evidence uncovered from the present study stand against such a claim: first of all, inhibition of TFEB activation by mTORC1 is through phosphorylation and cytoplasmic sequestration of TFEB, but our data show that the nuclear fraction of both isoforms of TFEB is increased in the R120G hearts at 6 m; secondly, increased mTORC1 activity at the earlier stage (1 m) of this disease model was not co-existed with a decrease in TFEB activity or decreased autophagic flux. Hence, it is very likely that factors other than mTORC1 may have come into play. Indeed, some of the neurodegenerative disease-associated proteins have been shown to directly interfere with TFEB’s transactivation activity by affecting both its nuclear translocation and actions in the nucleus (Bajaj et al., 2019). For example, α-synuclein was found to behave as a TFEB-sequestering molecule to accumulate TFEB in the Lewy bodies, thereby hindering TFEB from nuclear translocation in dopaminergic neurons of Parkinson’s disease (Decressac et al., 2013); and apolipoprotein E4 (apoE4), which is encoded by the *APOE* ε4 allele, the single greatest risk factor for Alzheimer’s disease in humans, was shown to compete with TFEB in binding to the CLEAR motif in the promoter of lysosomal genes (Parcon et al., 2018). It will be important to determine whether and how misfolded proteins such as CryAB^R120G^ would directly interfere with TFEB activity in cardiomyocytes as this is expected to shine a light on how to reactivate endogenous TFEB to protect against cardiac IPTS.

## Data Availability

All datasets generated for this study are included in the manuscript and/or the supplementary files.

## Author Contributions

XW contributed to conception and experimental design of the study, data analysis and interpretation, and manuscript preparation. BP contributed to data collection, analysis, interpretation, and manuscript preparation. ML helped in mouse genotyping and tissue collection, assistance in experimental design and preparation of sections of the manuscript. PW contributed to data collection and analyses.

### Conflict of Interest Statement

The authors declare that the research was conducted in the absence of any commercial or financial relationships that could be construed as a potential conflict of interest.
